# A unified nonlinear stochastic time series analysis for climate science

**DOI:** 10.1038/srep44228

**Published:** 2017-03-13

**Authors:** Woosok Moon, John S. Wettlaufer

**Affiliations:** 1 Institute of Theoretical Geophysics, Department of Applied Mathematics and Theoretical Physics, Centre for Mathematical Sciences, Wilberforce Road, Cambridge, CB3 0WA, United Kingdom; 2 Yale University, New Haven, USA; 3 Mathematical Institute, University of Oxford, Oxford, UK; 4 Nordita, Royal Institute of Technology and Stockholm University, SE-10691, Stockholm, Sweden

## Abstract

Earth’s orbit and axial tilt imprint a strong seasonal cycle on climatological data. Climate variability is typically viewed in terms of fluctuations in the seasonal cycle induced by higher frequency processes. We can interpret this as a competition between the orbitally enforced monthly stability and the fluctuations/noise induced by weather. Here we introduce a new time-series method that determines these contributions from monthly-averaged data. We find that the spatio-temporal distribution of the monthly stability and the magnitude of the noise reveal key fingerprints of several important climate phenomena, including the evolution of the Arctic sea ice cover, the El Ni
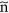
o Southern Oscillation (ENSO), the Atlantic Ni
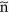
o and the Indian Dipole Mode. In analogy with the classical destabilising influence of the ice-albedo feedback on summertime sea ice, we find that during some time interval of the season a destabilising process operates in all of these climate phenomena. The interaction between the destabilisation and the accumulation of noise, which we term the *memory effect*, underlies phase locking to the seasonal cycle and the statistical nature of seasonal predictability.

The latitudinal and seasonal distribution of short-wave radiance reaching Earth’s surface drives the poleward atmospheric and oceanic transport of excess energy^1^,^2^,^3^ and generates the largest periodic signal observed in most monthly-averaged climate data. Climate variability can thus be viewed in terms of the statistical coupling between a range of non-seasonal spatio-temporal processes and the seasonal cycle of the energy flux balance. The manner in which this interplay operates depends on the latitude, the surface characteristics (land, ocean or ice-covered), and the flow of the atmosphere and the ocean. For example, in the tropics large-scale convective motions driven by the distribution of short-wave radiative flux and land-sea contrast underlie the Monsoon^4^ and the Intertropical Convergence Zone (ITCZ)^5^, which shape the dominant aspects of the seasonal cycle. In the mid-latitudes, synoptic-scale wave activity–the basic dynamics of weather–is controlled by the large-scale meridional temperature gradient between low- and high-latitudes^6^,^7^. Finally, in the polar regions the seasonal changes in the sea ice cover represent the key coherent signal of the seasons^8^.

Despite the geographical differences in the detailed dynamics of the seasonal cycle, there is a common feature; the seasonal cycle (or deterministic backbone) is driven by the externally-forced energy flux balance, whereas variability is associated with the interactions between higher frequency processes (“noise”) and the seasonal backbone. The strength of the seasonal variance is determined by the magnitude of the noise and the degree of seasonal (in)stability, as the seasonal (in)stability determines the degree of the (amplification) suppression of the noise. Thus, quantifying seasonal variability requires understanding this interaction.

The seasonal cycle itself is a combination of a myriad of physical processes, from radiative to fluid dynamical, that are formidable to deconvolve and isolate. Moreover, all sources of noise cannot be discerned. Our approach is to use a stochastic model that treats the monthly stability as the deterministic backbone influenced by noise forcing, and we extract these contributions from monthly-averaged climate data with our newly-developed time-series analysis. Using data spanning decades to centuries, we deduce the monthly stability, stochastic noise and decadal forcing for the Arctic sea ice cover, El Niño, the Atlantic Niño and the Indian Ocean Dipole.

The seasonal variability of Arctic sea ice is controlled by a deterministic backbone with a distinct two-season structure; the destabilising ice-albedo feedback operating in the summer and the stabilising longwave-loss of energy from the surface operating in the winter^9^,^10^. Similarly, the spatial distribution of the monthly stability reveals a destabilising process from July to November in the eastern tropical Pacific, the centre of ENSO, and stabilising processes during the rest of the year. This two-season structure may underlie ENSO’s phase locking to the seasonal cycle and can be generalised to explain the phase locking in the other phenomena we study here. Moreover, the destabilising component of the seasonal structure can be associated with the seasonal predictability barrier in Arctic sea ice and ENSO.

## Theoretical Approach

In the spirit of deterministic energy balance models^11^, consider the fate of the surface air temperature, *T*, evolving in response to seasonal forcing, *Q(T, t*), and decadal forcing, *F(τ*), according to 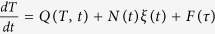, where the stochastic noise forcing, *N(t)ξ(t*), can be interpreted as the effect of high frequency weather-related contributions to the surface energy flux balance. If the climatological seasonal cycle of temperature, 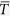, satisfies 
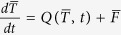
, then 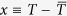 will approximately satisfy 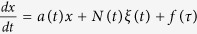, where 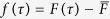 and 
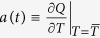
 is a periodic function capturing the stability (positive/negative) of the seasonal backbone. The magnitude of the noise *N(t*) is also a periodic function, modelling the seasonal cycle of the high-frequency processes, where *ξ(t*) is white noise defined by 〈*ξ(t)ξ(t*′)〉 = *δ(t* − *t*′). Therefore, this stochastic model of the temperature deviation *x* is a *non-autonomous* Langevin equation forced by a low frequency influence *f(τ*). Our method (see Methods and Supplementary Information) enables us to construct the periodic functions for the stability, *a(t*), the noise, *N(t*), and the low frequency forcing, *f(τ*), from observations and to thereby generate consistent stochastic dynamics of subsystems in the climate. Importantly, whilst we can construct these dynamics from the observations, our key results on the seasonal predictability barrier and phase locking are *independent* of the low frequency forcing *f(τ*). Therefore, the simplest stochastic model for a general climate variable *x(t*) that embodies the relationship between monthly stability and high frequency processes is


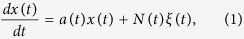


which is simply that described above with *f(τ*) = 0. The solution of equation (1) is a Gaussian


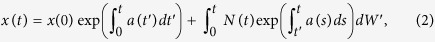


with *ξ(t*) = *dW*′/*dt*, and a variance of 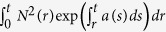, showing the statistical linkage between the deterministic dynamics and the noise. When *a(t*) is positive (negative) during some part of the year, fluctuations can grow (decay), thereby influencing the degree of variability as described in detail in ref. 10. Importantly, we note that the general approach of treating climate problems in a stochastic framework has a long history with many applications and foci^12^,^13^,^14^,^15^,^16^,^17^,^18^,^19^,^20^,^21^,^22^,^23^. Here, equation (1) is an extension of the well-known Ornstein-Uhlenbeck process to a *non-autonomous* periodic system and in what follows we (a) obtain analytical expressions for the time dependent coefficients, (b) extract their values from observations and thereby (c) demonstrate the veracity of using a one-dimensional approach in a broad range of climate processes.

As noted above, an important example of this two-season response is found in Arctic sea ice. Growth of fluctuations is associated with the sea-ice albedo feedback during summer^24^,^25^,^26^, whereas during winter, in the absence of solar insolation, *a(t*) becomes negative due to the stabilising influence of the longwave radiative loss from the surface^9^,^10^. Such fluctuation-feedbacks manifest themselves in a number of important climate subsystems with two-season structure. Next we demonstrate our method using two sets of gridded data; the Goddard Institute for Space Studies (GISS) surface temperatures from 1880 to 2012^27^,^28^, and the NOAA OI SST V2 monthly sea surface temperatures from 1981 to 2016^29^.

**Figure 1:**
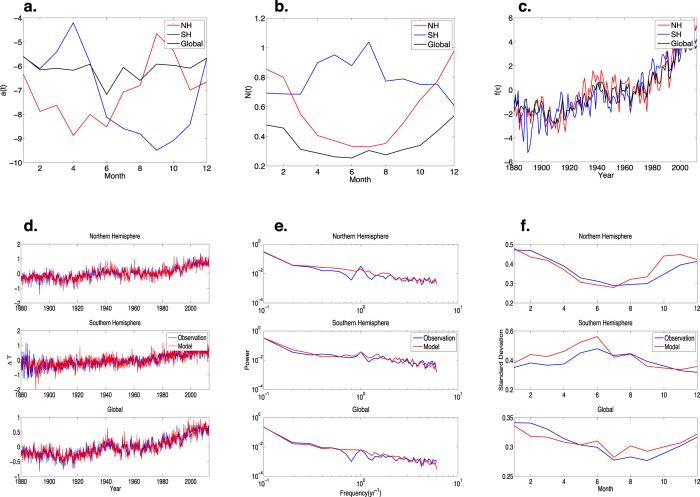
We apply our time-series method to 133 years of monthly averaged GISS surface air temperature data from 1881 to 2013^27^,^28^. We analyze the global (black), Northern hemisphere (red) and Southern hemisphere (blue) averaged data and determine the (**a**) monthly stability *a(t*), (**b**) noise intensity *N(t*), and (**c**) long-term forcing *f(τ*) from the model 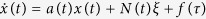. The model (red) is compared with the original time-series (blue) in (**d**) and via spectral power in (**e**). The spectral power of the stochastic model (red) and the data (blue) is calculated with Welch’s power spectral density estimate using 10-yr window size. Finally, in (**f**) we compare the seasonal standard deviation of the stochastic model (red) and the original data (blue). The overall comparison is good. The model assumptions that the surface energy flux balance can be considered as originating from weather, seasonal and decadal processes is well born out by the analysis. The high-frequency weather contribution is represented as the noise forcing *N(t)ξ(t*), wherein the intensity *N(t*) is related to the seasonal cycle of baroclinicity, being larger (smaller) winter (summer). The seasonality of the monthly stability *a(t*) is associated with that of the seasonality of the insolation. The September Northern hemisphere maximum of *a(t*) is commensurate with the minimum Arctic sea ice extent. Finally, the long-term forcing *f(τ*) is responsible for decadal variability, but as we see below, this contribution is not essential for understanding seasonal variability and predictability barriers and phase locking.

The global (black), northern hemisphere (red) and southern hemisphere (blue) averaged GISS values of *a(t*), *N(t*) and *f(τ*) constructed by the method are shown in Fig. 1(a–c), respectively. Using these values we generate a stochastic realisation of equation (1) with *f(τ*), and plot it (red) with the original time series (blue), the power spectra, and the seasonal standard deviations in Fig. 1(d–f) respectively. This clearly shows that the two time-series evolve in a statistically similar manner and in particular that the model captures the observed seasonal variability.

**Figure 2:**
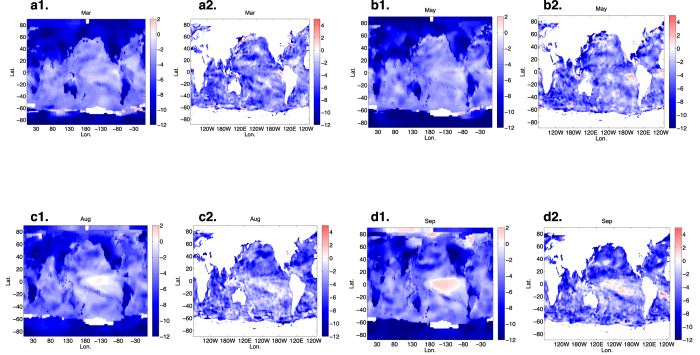
The seasonal cycle of the monthly stability *a(t*) as a function of latitude and longitude. In (**a1**,**b1**,**c1** and **d1**) we plot the values of *a(t*) determined from 133 years of monthly averaged GISS surface air temperature data from 1881 to 2013 discussed in Fig. 1 and in (**a2**,**b2**,**c2** and **d2**) we plot the values of *a(t*) determined from 36 years of NOAA OI SST V2 data from 1981 to 2016. We observe a striking seasonal evolution in the eastern tropical Pacific, which is particularly strong in the longer GISS record. From January to June a strong stabilising process is operative and is reflected in the negative values of *a(t*), shown here for March (**a1**,**a2**) and May (**b1**,**b2**). In contrast, from July to December the values of *a(t*) change sign, signaling a destabilising process, shown here for August (**c1**,**c2**) and September (**d1**,**d2**). This demonstrates that in the eastern tropical Pacific the stability exhibits a two-season structure, which is a key ingredient in ENSO’s phase locking to the seasonal cycle. Note that, as always included in reanalysis data, in (**a2**,**b2**,**c2** and **d2**), we used the land masked data, with 0 (1) over ocean (land), as described at http://www.esrl.noaa.gov/psd/repository/entry/show?entryid=b5492d1c-7d9c-47f7-b058-e84030622bbd.

Figure 2 shows the global spatial distribution of the *a(t*) for March (a1 and a2), May (b1 and b2), August (c1 and c2) and September (d1 and d2), with the GISS and NOAA data labeled 1 and 2 respectively. We draw the readers attention to the eastern tropical Pacific Ocean where we see that the *a(t*) are negative during March and May and positive during August and September. In the complete time series (a subset of which are shown in Fig. 2) we find that from December to June, the values of *a(t*) in the eastern tropical Pacific are negative, indicating the existence of stabilising processes, whereas from July to November the values of *a(t*) are positive, indicating the dominance of destabilising processes. Therefore, the data show that ENSO has the same two-season stability structure as does Arctic sea ice, suggesting the same general theoretical framework may be applicable to their seasonal variability. In the Supplementary Information we show why our approach reproduces that of the two-dimensional recharge oscillator model^17^,^30^.

## The Memory Effect, Seasonal Variability & Phase Locking

**Figure 3:**
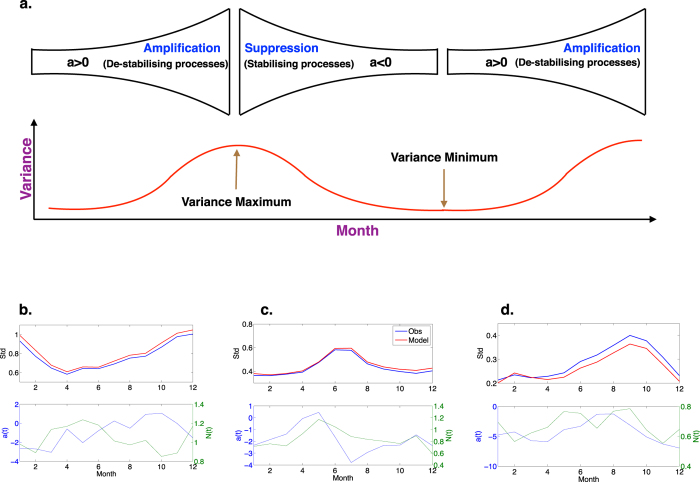
A schematic representing the seasonal evolution of the variance with the two-season stability structure (**a**). During the period in which *a(t*) is positive destabilising processes dominate the dynamics, amplifying fluctuations, *N(t)ξ(t*). As *a(t*) changes sign stabilising processes dominate, suppressing the fluctuations. There are two transition points between them. When *a(t*) is positive (negative) noise generated variability is accumulated (suppressed) up to the transition point. Hence, the maximum variance occurs at the transition point from positive to negative stability. We show this dynamics using our time series analysis on three major climate indices; the Nino3 SST index (**b**), the Atlantic Niño index (**c**) and the Dipole Mode Index (**d**). In each pair of panels we plot on the top the monthly standard deviation calculated from the data (blue line) and from the stochastic model of equation 1 (red line), and in the bottom panel the *a(t*) (blue line) and *N(t*) (green line) constructed by our time series method. All indices exhibit a variance maximum proximal to the transition from destabilising to stabilising dynamics and the stochastic model produces monthly standard deviations in excellent agreement with the observations alone without the need to include long-term variability, *f(τ*). Therefore, it appears that the monthly stability *a(t*) and the accumulation of noise–memory effect–are sufficient to capture phase locking as observed in seasonal climate dynamics.

The generic behavior of two-season dynamics is embodied in a “memory effect” that we have described for Arctic sea ice^10^. Because of a separation of time scales, the long term forcing does not play a role here and thus we examine the consequences of equation (1), whose solution (Eq. 2) and its variance are represented by a delayed-integral, which weds the monthly stability *a(t*) to the noise magnitude *N(t*). This marriage explains how a fluctuation in a given–destabilising or stabilising–season is suppressed or amplified over time. Therefore, it matters *when* in the seasonal cycle a fluctuation occurs, with the extremes in the associated variance manifesting themselves at the two transition points where the stability changes sign. For example, in the case of Arctic sea ice *a(t*) changes sign from destabilising to stabilising (stabilising to de-stabilising) near the end of summer (winter), and this is reflected as a maximal (minimal) variance in September (March). In the case of fluctuations of surface air and sea surface temperatures in the eastern tropical Pacific, the variance is maximal near the end of year (December) and minimal near the early summer (June). Importantly we note that the variance is not maximised when the deterministic destabilising processes are maximised (i.e., when |*a(t*)| is maximal). Rather, the influence of destabilising processes accumulates over time until the point at which destabilisation gives way to stabilisation. This “memory effect” is described in Fig. 3(a). Next we show how two-season characteristics and the memory effect underlie the seasonal variability for a troika of climate indexes from the tropical oceans.

The results from (i) the Nino3 SST index (the area averaged SST from 5 S-5 N and 150 W-90 W), representing the intensity of ENSO^31^, (ii) the Atlantic Niño index^32^,^33^ (the area averaged SST from 3 S-3 N and 15 W-0 W), and (iii) the Dipole Mode Index^34^, which is the anomalous SST gradient between the western equatorial Indian Ocean and the south eastern equatorial Indian Ocean, are shown in Fig. 3(b–d) respectively. In the upper panels the standard deviation calculated from these three data sets is compared to that from the stochastic model in equation (1), where the latter uses the stability, *a(t*), and the noise, *N(t*), constructed from the data as shown in the lower panels, all of which show two-season dynamics. The values of *a(t*) for the Nino3 SST index are positive from July to November creating a maximal variance as the end of the year is approached. The values of *a(t*) for the Atlantic Niño Index change sign, from positive to negative, in May resulting in a maximal variance in the boreal summer. Although the *a(t*) values for the Dipole Mode Index are always negative, because the magnitude of the stability increases (i.e., it is more negative) from August to December, we see a maximal variance in September. We note that the seasonal cycle of *a(t*) for the Nino3 SST index is consistent with previous work that emphasises the role of the seasonality of the background stability–the seasonal Bjerknes instability index^30^,^35^,^36^. Importantly, the variance produced by the stochastic model (equation 1) without long term forcing captures the behavior of the data, showing that the combination of two-season dynamics and the memory effect explain the seasonal cycle of the variance of these climate indexes, and the examples discussed above.

The observation that El Niño and La Niña events are principally concentrated in November or December^37^ is referred to as the “phase-locking” of ENSO to the seasonal cycle^38^. The peak phase of the Atlantic Niño occurs approximately in the boreal summer^39^ and that of the Indian Dipole occurs approximately in October^34^. The seasonal variance of the state of the Arctic sea ice cover is maximal at the end of the summer^10^. We consider these phenomena to be general examples of “seasonal phase locking”. For example, from the statistical perspective, El Niño and La Niña represent conditions far from the mean state of the ENSO signal. Hence the question of why El Niño and La Niña tend to occur far more frequently near the end of a year is equivalent to asking why the probability of the seasonal occurrence of these events is higher near the end of a given year. In other words, why does the maximal ENSO variance occur at the end of the year? In this sense, the timing of the maximal seasonal variance of all of these phenomena can be considered to be “seasonal phase locking”. Underlying this is the two-season dynamics described by the stability transition (*a(t*) changing from destabilising to stabilising) where the accumulation of fluctuations–the memory effect–leads to a maximum in the variance.

## Seasonal predictability barrier

The Arctic sea ice cover and ENSO are phenomena of intense focus with regards to seasonal predictability, with sharp declines in the former after a few months^25^,^40^,^41^,^42^ and in the latter during the boreal spring–the spring predictability barrier^43^,^44^. As described above, both systems possess two-season characteristics as reflected in the monthly stability. Now we pursue their relationship to seasonal predictability barriers.

The strong two-season structure of Arctic sea ice is roughly characterized in terms of summer and winter^9^. As the system enters into the summer season and the destabilising sea ice-albedo feedback becomes operative, and natural fluctuations or model errors–noise–are magnified^10^. This amplification of fluctuations underlies the loss of predictability at the end of the melt season. On the other hand, fluctuations that arise during the fall freeze up are highly suppressed by the stabilising influence of the surface long-wave energy loss^10^. The albedo feedback amplification is the key aspect of the seasonal predictability barrier for Arctic sea ice.

**Figure 4:**
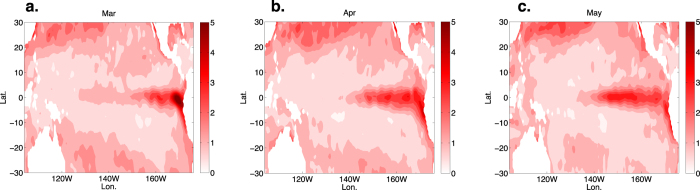
The noise intensity *N*(*t*) constructed from monthly NOAA OI SST V2 data from December 1981 to January 2016 available at http://www.esrl.noaa.gov/psd/repository/entry/show?entryid=b5492d1c-7d9c-47f7-b058-e84030622bbd, as also described in Fig. 2. We see that during March (**a**), April (**b**) and May (**c**), a “tongue” of *N(t*) grows from the boundary of eastern tropical Pacific (which may be related to coastal upwelling) and then extends toward the centre of tropical Pacific. During the same period the monthly stability *a(t*) is negative in the eastern tropical Pacific, indicating the dominance of stabilising processes.

The ENSO spring predictability barrier refers to the fact that most models show a decline in forecasting skill from March to May^44^, and data show a decline in monthy “persistence”^45^. In Fig. 4 we show the March to May values of *N(t*) in the tropical Pacific constructed from NOAA OI SST v2 monthly sea surface temperature data. During this period, Fig. 4(a–c) show that the background noise grows from the Peruvian coastal region into the central tropical Pacific. Moreover, *a(t*) is negative and *N(t*) is large in equation (1). The first term of the stochastic solution (equation 2) shows the contribution of the initial condition and the second term shows the contribution of the noise. During the spring the large negative values of *a(t*) insure a negligible contribution of the initial condition so that the noise term dominates. Indeed, during the spring the SST anomaly (typically represented by the NINO 3 index) is principally controlled by high frequency processes in the eastern tropical Pacific. Due to the difficulty of simulating high frequency processes in climate models, model errors are more likely to be present during this time period, and will thus be magnified by the de-stabilising processes that start in July and thereby degrade model predictability. Therefore, the essence of ENSO’s spring predictability barrier is the generation of errors during the boreal spring and their subsequent amplification beginning in the summer and extending through the remainder of the year.

The “persistence” is defined as the fixed phase correlation between different months, and the “persistence barrier” reflects the seasonal predictability barrier of ENSO^45^. Torrence and Webster (Ref. 45) showed from observations that regardless of starting month, the persistence drops sharply from March to May, suggesting a link to the seasonal evolution of the persistence in our stochastic framework as follows. The persistence of equation (1) is 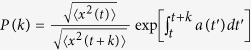 and is controlled by the ratio of the variance of the initial month *t* to that of any final month *t* + *k*, and by the time-integral of the stability *a(t*) over the interval *k*. Thus, the sign and magnitude of the stability *a(t*) plays a crucial role. Indeed, the strongly negative values of *a(t*) in the eastern tropical Pacific imply a sharp decline of the persistence from March to May. Moreover, during the same period, the substantial increase in the noise *N(t*) shown in Fig. 4 leads to a substantial increase in 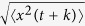, also implying a decrease in persistence.

**Figure 5:**
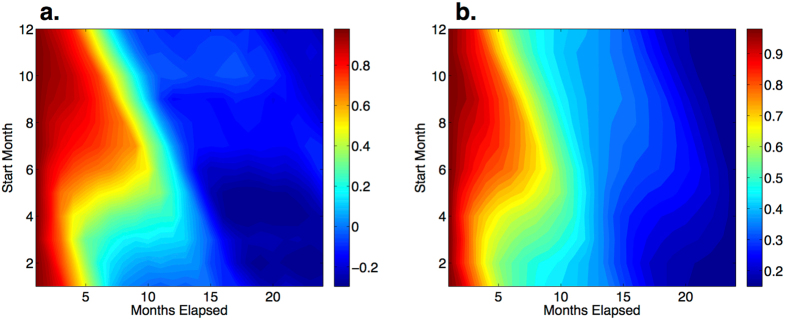
Persistence contours with the *y*-axis the start month and the *x*-axis the months elapsed. The persistence from the original Nino3 SST index, from 1870 to 2015 (**a**), and for comparison in (**b**) we show the associated results from equation 1. Note, even without the inclusion of the long-term forcing *f(τ*), the persistence calculated using the stochastic model is consistent with that from the original Nino3 index data. Therefore, the seasonal variability reflected in the persistence is well approximated solely by the monthly stability *a(t*) and the noise intensity *N(t*). In particular, regardless of the starting month, in both figures we see that the persistence drops sharply from March to May.

Having applied our time-series method to the Nino3 SST index^31^ (Fig. 3b), for a range of initial months we calculate the persistence from the data (Fig. 5a) and from the stochastic model equation (1) (Fig. 5b). Both approaches exhibit the main characteristic of a sharp decline in the persistence from March to May, only weakly dependent on the starting month. Hence, the confluence of negative stability and an increase in statistical noise during the boreal spring combine to decrease the persistence and the predictability.

Clearly, reducing or removing the model based predictability barriers in Arctic sea ice and ENSO requires reliable representations of high frequency processes and the destablising mechanisms amplifying them. The challenges are substantial. In the former case, the destabilising summer period where the ice-albedo feedback is operative involves capturing melt pond evolution, mechanical deformation, cloud forcing, ice-ocean heat fluxes and other processes that modify the energy balance on short time scales. In the latter case, the key positive feedback is the Bjerknes feedback^46^, embodying large-scale tropical convective processes associated with the monsoon^47^ and the Intertropical Convergence Zone (ITCZ)^48^, and their interaction with ocean upwelling^49^.

## Conclusions

Since the introduction of a simple Ornstein-Uhlenbeck process into climate research^12^ it is often used to analyse observations in terms of a single decay time, or as a red-noise process. By generalising the stochastic approach to the periodic non-autonomous model of equation (1), many key features of the seasonal variability of climate subsystems are captured. We find that the periodicity of the stability, *a(t*), and its interaction with the noise, *N(t*), reveals a detailed picture of seasonal variability. The framework leads to a method of time series analysis that allows us to construct these quantities, in addition to long-term forcing effects *f(τ*) (although this turns out to be superfluous for the phenomena we examine here), shows that this stochastic model captures the behaviour of a wide range of climate data.

The main dynamics is as follows. The two-season structure of these climate subsystems is represented by the annual oscillations (principally with changes in sign) of the stability. The nature of the accumulation of noise in the stable/unstable part of the cycle is referred to as the “memory effect”. These basic processes allow us to explain the seasonal phase locking of Arctic sea ice, ENSO, the Atlantic Niño and the Indian Dipole Oscillation. Important events frequently occur at the transition between destabilising (*a(t*) > 0) and stabilising (*a(t*) < 0) seasonal states. This underlies phase locking and seasonal predictability barriers, in which fluctuations (or model errors) are amplified in the destabilising part of the two-season structure and thus achieve a maximal effect by the time the transition is reached. Therefore, the time-dependent interaction between the background stability and the noise constitute a minimal description of seasonal climate dynamics. We suggest that by deliberate manipulation of the high frequency dynamics (e.g., by filtering or time dependent forcing), these ideas can be tested in a climate model setting where stochastic processes are now being shown to play a central role^50^.

## Methods

In our time-series analysis we construct the monthly stability, *a(t*), the noise intensity, *N(t*), and the long-term forcing, *f(τ*), from a monthly-averaged climate variable 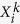, where *k* and *i* represent the month and year, respectively. The theoretical background for this time-series analysis method, quoting here only the results, is detailed in the Supplementary Information.

### Calculation of the basic statistics of a time series 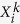


The basic statistics include the monthly variance, *S(k*), the correlation between two adjacent months, *A(k*), and the one or two year auto-correlation, *B(k*), which are


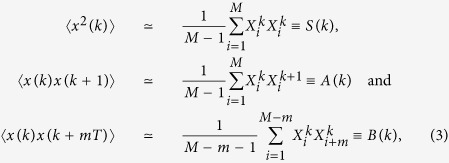


where *x(k*) is the solution of the stochastic differential equation. We have written this for the *k*th month where *T* = 1*yr* and here Δ*t* = 1 month so *k* + Δ*t* = *k* + 1. We see that for *B(k*) we can choose *m* to be 1 or 2 accordingly. All of these quantities are periodic functions with with a period of 1*yr*. These expressions are derived in the Supplementary Information.

### Monthly stability *a(t*)

Here we let


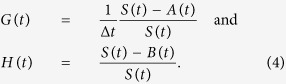


We then solve the periodic non-autonomous ordinary differential equation, *dP*/*dt* = −*G(t)P(t*) + *H(t*), to find the steady-state periodic function *P(t*). The monthly stability is then *a(t*) = 1/*P(t*) × [*H(t*) − *G(t)P(t*) − 1].

### Noise intensity *N(t*)

First we construct a new data set from the original data viz., *y(t*) = *x(t* + Δ*t*) − *x(t*) − *a(t)x(t*)Δ*t* using the *a(t*) from the previous step. We observe that 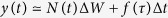, where Δ*W* is a Brownian time step, which leads to


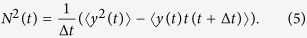


### Long-term forcing *f(τ*)

At this stage we know *a(t*) and *N(t*), so all that remains is to determine *f(τ*) from the model equation *x(t* + Δ*t*) − *x(t*) − *a(t)x(t*)Δ*t* − *N(t*)Δ*W*. Because *f(τ*) represents decadal scale forcing, and hence has a characteristic time scale much longer than seasonal, we assume it takes only one value per year. Finally, we take the annual average of the residual as


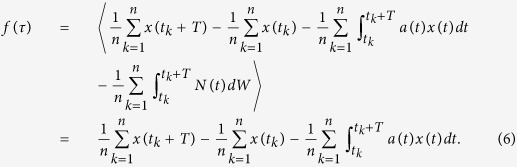


## Additional Information

**How to cite this article:** Moon, W. and Wettlaufer, J. S. A unified nonlinear stochastic time series analysis for climate science. *Sci. Rep.* **7**, 44228; doi: 10.1038/srep44228 (2017).

**Publisher's note:** Springer Nature remains neutral with regard to jurisdictional claims in published maps and institutional affiliations.

## Supplementary Material

Supplementary Information

## References

[b1] Houghton H. G. On the Annual Heat Balance of the Northern Hemisphere. J. Meteor. 11 **(1)**, 1–9 (1954).

[b2] Manabe S. Climate and the ocean circulation 1: 1. The atmospheric circulation and the hydrology of the Earth’s surface. Mon. Wea. Rev. 97 **(11)**, 739–774 (1969).

[b3] Bryan K., Manabe S. & Pacanowski R. C. A global ocean-atmosphere climate model. Part 2. The ocean circulation. J. Phys. Oceanogr. 5 **(1)**, 30–46 (1975).

[b4] Webster P. J. . Monsoons: Processes, predictability, and the prospects for prediction. J. Geophys. Res-Oceans 103 **(C7)**, 14451–14510 (1998).

[b5] Waliser D. E. & Gautier C. A satellite-derived climatology of the ITCZ. J. Climate 6 **(11)**, 2162–2174 (1993).

[b6] Charney J. G. The dynamics of long waves in a baroclinic westerly current. J. Meteor. 4 **(5)**, 136–162 (1947).

[b7] Eady E. T. Long waves and cyclone waves. Tellus 1 **(3)**, 33–52 (1949).

[b8] Budikova D. Role of Arctic sea ice in global atmospheric circulation: A review. Glob. Planet. Change 68 **(3)**, 149–163 (2009).

[b9] Moon W. & Wettlaufer J. S. A low-order theory of Arctic sea ice stability. EPL 96 **(3)**, 39001 (2011).

[b10] Moon W. & Wettlaufer J. S. A stochastic perturbation theory for non-autonomous systems. J. Math. Phys. 54 **(12)**, 123303 (2013).

[b11] North G. R., Cahalan R. F. & Coakley J. A. Jr Energy balance climate models. Rev. Geophys. 19 **(1)**, 91–121 (1981).

[b12] Hasselmann K. Stochastic climate models part 1. Theory. Tellus 28 **(6)**, 473–485 (1976).

[b13] North G. R. & Cahalan R. F. Predictability in a solvable stochastic climate model. J. Atmos. Sci. 38 **(3)**, 504–513 (1981).

[b14] Benzi R., Parisi G., Sutera A. & Vulpiani A. Stochastic resonance in climate change. Tellus 34 **(1)**, 10–16 (1982).

[b15] Messi P. A simple box model of stochastically forced thermohaline flow. J. Phys. Oceanogr. 24 **(9)**, 1911–1920 (1994).

[b16] Penland C. & Sardeshukh P. D. The optimal growth of tropical sea surface temperature anomalies J. Climate 54 **(7)**, 811–829 (1995).

[b17] Jin F. F. An equatorial ocean recharge paradigm for ENSO. Part 1: Conceptual model. J. Atmos. Sci. 54 **(7)**, 811–829 (1997).

[b18] Tziperman E., Raymo M. E. Huybers P. & Wunsch C. Consequences of pacing the Pleistocene 100 kyr ice ages by nonlinear phase locking to Milankovitch forcing. Paleoceanography 21, PA4206, doi: 10.1029/2005PA001241 (2016).

[b19] Majda A. J., Franzke C. & Khouider B. An applied mathematics perspective on stochastic modelling for climate. Philos. Trans. Roy. Soc. 366A, 2429–2453 (2008). 10.1098/rsta.2008.0012 18445572

[b20] Chekroun M. D., Simonnet E. & Ghil M. Stochastic climate dynamics: Random attractors and time-dependent invariant measures. Physica D 240 **(21)**, 1685–1700 (2011).

[b21] Dijkstra H. Nonlinear Climate Dynamics (Cambridge University Press, Cambridge, England, 2013).

[b22] Kondrashov D. & Berloff P. Stochastic modeling of decadal variability in ocean gyres. Geophys. Res. Lett. 42 1543–1553 (2015).

[b23] Franzke C., O’Kane T. J., Berner J., Williams P. D. & Lucarini V. Stochastic climate theory and modeling. WIREs Clim Change 6, 63–78 (2015).

[b24] Maykut G. A. & Untersteiner N. Some results from a time dependent thermodynamic model of sea ice. J. Geophys. Res. 76, 1550–1575 (1971).

[b25] Schroeder D., Feltham D. L., Flocco D. & Tsamados M. September Arctic sea-ice minimum predicted by spring melt-pond fraction. Nat. Clim. Change 4, 353–357 (2014).

[b26] Webster M. A. . Seasonal evolution of melt ponds on Arctic sea ice. J. Geophys. Res.-Oceans 120 5968–5982 (2015).

[b27] GISTEMP Team, GISS Surface Temperature Analysis (GISTEMP). NASA Goddard Institute for Space Studies. Dataset accessed 2016-01-15 at http://data.giss.nasa.gov/gistemp/ (2016).

[b28] Hansen J. E., Ruedy R., Sato M. & Lo K. Global surface temperature change. Rev. Geophys. 48, RG4004, doi: 10.1029/2010RG000345 (2010).

[b29] Reynolds R. W., Rayner N. A., Smith T. M., Stokes D. C. & Wang W. An improved *in situ* and satellite SST analysis for climate. J. Climate 15, 1609–1625 (2002).

[b30] Levine A. F. & McPhaden M. J. The annual cycle in ENSO growth rate as a cause of the spring predictability barrier. Geophys. Res. Lett. 42 **(12)**, 5034–5041 (2015).

[b31] Rayner N. A. . Global analyses of sea surface temperature, sea ice, and night marine air temperature since the late nineteenth century J. Geophys. Res. 108 **(D14)**, 4407 (2003).

[b32] Lutz K., Rathmann J. & Jacobeit J. Classification of warm and cold water events in the eastern tropical Atlantic Ocean. Atmos. Sci. Lett. 2, 102–106 (2013).

[b33] Lutz K., Rathmann J. & Jacobeit J. Atlantic warm and cold water events and impact on African west coast precipitation. Int. J. Climatol. 35 **(1)**, 128–141 (2014).

[b34] Saji N. H., Goswami B. N., Vinayachandran P. N. & Yamagata T. A dipole mode in the tropical Indian Ocean. Nature 401 **(6751)**, 360–363 (1999). 16862108 10.1038/43854

[b35] Stein K., Schneider N., Timmermann A. & Jin F. F. Seasonal synchronisation of ENSO events in a linear stochastic model. J. Climate 23 **(21)**, 5629–5643 (2010).

[b36] Stein K., Timmermann A., Schneider N., Jin F. F. & Stuecker M. F. ENSO seasonal synchronisation theory. J. Climate 27 **(14)**, 5285–5310 (2014).

[b37] An S. I. & Wang B. Mechanisms of locking of the El Niño and La Niña mature phases to boreal winter. J. Climate 14 **(9)**, 2164–2176 (2001).

[b38] Tziperman E., Cane M. A. & Zebiak S. E. Irregularity and locking to the seasonal cycle in an ENSO prediction model as explained by the quasi-periodicity route to chaos. J. Atmos. Sci. 52 **(3)**, 293–306 (1995).

[b39] Zebiak S. E. Air-sea interaction in the equatorial Atlantic region. J. Climate 6 **(8)**, 1567–1586 (1993).

[b40] Lindsay R. W., Zhang J., Schweiger A. J. & Steele M. A. Seasonal predictions of ice extent in the Arctic Ocean. J. Geophys. Res.-Oceans 113 **(C2)** (2008).

[b41] Blanchard-Wrigglesworth E., Armour K. C., Bitz C. M. & DeWeaver E. Persistence and inherent predictability of Arctic sea ice in a GCM ensemble and observations. J. Climate 24 **(1)**, 231–250 (2011).

[b42] Wang W., Chen M. & Kumar A. Seasonal prediction of Arctic sea ice extent from a coupled dynamical forecast system. Mon. Wea. Rev. 141 **(4)**, 1375–1394 (2013).

[b43] Latif M. . A review of ENSO prediction studies. Clim. Dynam. 9 **(4–5)**, 167–179 (1994).

[b44] Webster P. J. & Yang S. Monsoon and ENSO: Selectively interactive systems. Q. J. R. Meteorol. Soc. 118 **(507)**, 877–926 (1992).

[b45] Torrence C. & Webster P. J. The annual cycle of persistence in the El Niño/Southern Oscillation. Q. J. R. Meteorol. Soc. 124 **(550)**, 1985–2004 (1998).

[b46] Bjerknes J. A possible response of the atmospheric Hadley circulation to equatorial anomalies of ocean temperature. Tellus 18 **(4)**, 820–829 (1966).

[b47] Heddinghaus T. R. & Krueger A. F. Annual and interannual variations in outgoing longwave radiation over the tropics. Mon. Wea. Rev. 109 **(6)**, 1208–1218 (1981).

[b48] Horel J. D. On the annual cycle of the tropical Pacific atmosphere and ocean. Mon. Wea. Rev. 110 **(12)**, 1863–1878 (1982).

[b49] Mitchell T. P. & Wallace J. M. The annual cycle in equatorial convection and sea surface temperature. J. Climate 5 **(10)**, 1140–1156 (1982).

[b50] Palmer T. N. Towards the probabilistic Earth-system simulator: a vision for the future of climate and weather prediction. Q. J. R. Meteorol. Soc. 138 **(665)**, 841–861 (2012).

