# Supplementary material for: A unified nonlinear stochastic time series analysis for climate science
Source: Sci Rep. 2017 Mar 13;7:44228. doi: 10.1038/srep44228 (PMC5347016; doi:10.1038/srep44228)
Supplement: Supplementary Information [file srep44228-s1.pdf]

Supplementary Information: A unified nonlinear stochastic time series analysis for climate science.

W. Moon and J. S. Wettlaufer

I. FORMULATION OF THE PROBLEM

The seasonal cycle of surface temperature is principally controlled by the surface energy flux balance consisting of incoming shortwave radiance, outgoing and incoming longwave radiance, sensible and latent heat flux. The seasonality is clearly seen in observational data, but additionally, long-term monthly data contains high frequency fluctuations, or noise, and decadal time scales. Therefore, the energy flux balance can be written as

$$\rho_{air} c_p h_{air} \frac{dT}{dt} = Q^*(T,t) + N^*(t)\xi(t) + F^*(\tau), \tag{1}$$

where T is the surface temperature, $Q^*(T,t)$ represents the seasonal surface fluxes, $N(t)\xi(t)$ is the noise, with a magnitude, $N(t)$, that has a seasonal variation, and $f(\tau)$ represents long-term decadal forcing. The high frequency contribution to the noise $\xi(t)$ is assumed to be white viz., $\langle \xi(t)\xi(t')\rangle = \delta(t-t')$. The air density, heat capacity and the average depth of atmospheric boundary layer are ρ_{air}, c_p and h_{air}, respectively. To apply the stochastic model to monthly-averaged data, we scale time accordingly, with $\Delta t = 2.592 \times 10^6$s ~ 1 month. Hence,

$$\frac{dT}{dt} = \frac{\Delta t}{\rho_{air} c_p h_{air}} \left[Q^*(T,t) + N^*(t)\xi + F^*(\tau) \right], \tag{2}$$

which leads to

$$\frac{dT}{dt} = Q(T,t) + N(t)\xi + F(\tau), \tag{3}$$

where

$$\begin{aligned} Q(T,t) &= \frac{\Delta t}{\rho_{air} c_p h_{air}} Q^*(T,t), \\ N(t) &= \frac{\Delta t}{\rho_{air} c_p h_{air}} N^*(t) \quad \text{and} \\ F(\tau) &= \frac{\Delta t}{\rho_{air} c_p h_{air}} F^*(\tau). \end{aligned} \tag{4}$$

To determine the long-term variability in the monthly temperature data, we consider the deviation from an average seasonal cycle $\bar{T}(t)$, which satisfies

$$\frac{d\bar{T}}{dt} = Q(\bar{T},t) + \bar{F}(\tau). \tag{5}$$

Hence, if we let $T \equiv \bar{T} + x$ and $F \equiv \bar{F} + f$, x satisfies

$$\frac{dx(t)}{dt} = a(t)x(t) + N(t)\xi(t) + f(\tau), \tag{6}$$

where $t = \epsilon\tau$, with $\epsilon \ll 1$, and $a(t) \equiv \frac{\partial Q}{\partial T}|_{T=\bar{T}}$. The stochastic solution $x(t)$ is

$$\begin{aligned} x(t) &= \exp\left(\int_0^t a(r)dr\right) \int_0^t N(t')\exp\left(-\int_0^{t'} a(s)ds\right) dW' \\ &+ \exp\left(\int_0^t a(r)dr\right) \int_0^t f(\tau)\exp\left(-\int_0^{t'} a(s)ds\right) dt', \end{aligned} \tag{7}$$

where $\xi(t) = dW'/dt$. There are two time scales in equation (7); the decadal time scale, represented by τ, and the response time scale of the surface energy flux balance, $1/|\int_0^\Gamma a(r)dr| \equiv \mathcal{T}_a$, where Γ is 1-year. The latter time scale

is controlled by the monthly stability $a(t)$, which underlies our key approximation, $\mathcal{T}_a \ll 1/\epsilon$, so that equation (7) becomes

$$x(t) \simeq \exp\left(\int_0^t a(r)dr\right)\int_0^t N(t')\exp\left(-\int_0^{t'} a(s)ds\right)dW' + f(\tau)\exp\left(\int_0^t a(r)dr\right)\int_0^t \exp\left(-\int_0^{t'} a(s)ds\right)dt'. \tag{8}$$

Thus, equation (8) is the statistical model for monthly observations spanning decades, and our central problem is then to construct $a(t)$, $N(t)$ and $f(\tau)$ from the data.

II. DETERMINING $a(t)$, $N(t)$ AND $f(\tau)$ OF EQUATION (6)

A. Determination of $a(t)$.

In this section, we describe the theory developed to construct the three functions $a(t)$, $N(t)$ and $f(\tau)$ from equation (6) using monthly-averaged data. The variance follows from equation (8) as

$$\langle x^2(t)\rangle = \exp\left(2\int_0^t a(r)dr\right)\int_0^t N^2(t')\exp\left(-2\int_0^{t'} a(s)ds\right)dt' + \langle f^2\rangle\left[\exp\left(\int_0^t a(r)dr\right)\int_0^t \exp\left(-\int_0^{t'} a(s)ds\right)dt'\right]^2, \tag{9}$$

where $\langle\cdot\rangle$ is the time average. The main goal of our approach is to determine $a(t)$ and $N(t)$ from which we estimate the long-term slowly-varying forcing $f(\tau)$ as a residual. The central assumption is that the high-frequency processes approximated by $N(t)\xi(t)$ are stationary and the response time-scale $\mathcal{T}_a$ is much shorter than the total length of the data record.

The autocorrelation can be obtained from equation (8) as

$$\langle x(t)x(t+\Delta t)\rangle = \exp\left(\int_t^{t+\Delta t} a(r)dr\right)\exp\left(2\int_0^t a(r)dr\right)\int_0^t N^2(t')\exp\left(-2\int_0^{t'} a(s)ds\right)dt' + \langle f^2\rangle\exp\left(\int_0^{t+\Delta t} a(r)dr\right)\exp\left(\int_0^t a(r)dr\right) \times \int_0^{t+\Delta t}\exp\left(-\int_0^{t'} a(s)ds\right)dt'\int_0^t \exp\left(-\int_0^{t'} a(s)ds\right)dt', \tag{10}$$

where Δt is the basic time unit in which the data are measured. Hence, for monthly averaged data, Δt is a month. We can approximate

$$\int_0^{t+\Delta t}\exp\left(-\int_0^{t'} a(s)ds\right)dt' \simeq \int_0^t \exp\left(-\int_0^{t'} a(s)ds\right)dt' + \exp\left(-\int_0^{t+\Delta t} a(r)dr\right)\Delta t, \tag{11}$$

which allows us to rewrite equation (10) as

$$\langle x(t)x(t+\Delta t)\rangle = \exp\left(\int_t^{t+\Delta t} a(r)dr\right)\left[\exp\left(2\int_0^t a(r)dr\right)\int_0^t N^2(t')\exp\left(-2\int_0^{t'} a(r)dr\right)dt' + \langle f^2\rangle\left(\exp\left(\int_0^t a(r)dr\right)\int_0^t \exp\left(-\int_0^{t'} a(s)ds\right)dt'\right)^2\right] + \langle f^2\rangle\exp\left(\int_0^t a(r)dr\right)\int_0^t \exp\left(-\int_0^{t'} a(s)ds\right)dt'\Delta t. \tag{12}$$

Now, with the aide of equation (9), we can write

$$\langle x(t)x(t+\Delta t)\rangle = \exp\left(\int_t^{t+\Delta t} a(r)dr\right)\langle x^2\rangle + \langle f^2\rangle \Delta t \exp\left(\int_0^t a(r)dr\right)\int_0^t \exp\left(-\int_0^{t'} a(s)ds\right)dt'. \tag{13}$$

We define $P(t)$ as

$$P(t) \equiv \exp\left(\int_0^t a(r)dr\right)\int_0^t \exp\left(-\int_0^{t'} a(s)ds\right)dt', \tag{14}$$

which leads to

$$P(t+\Delta t) \simeq \exp\left(\int_t^{t+\Delta t} a(r)dr\right)P(t) + \Delta t, \tag{15}$$

and hence,

$$\langle x(t)x(t+\Delta t)\rangle = \frac{[P(t+\Delta t)-\Delta t]}{P(t)}\langle x^2\rangle + \langle f^2\rangle \Delta t P(t). \tag{16}$$

Now we approximate $\langle x^2(t)\rangle$ and $\langle x(t)x(t+\Delta t)\rangle$ from the time series data as

$$\langle x^2(k)\rangle \simeq \frac{1}{M-1}\sum_{i=1}^{M} X_i^k X_i^k \equiv S(k)$$
$$\langle x(k)x(k+1)\rangle \simeq \frac{1}{M-1}\sum_{i=1}^{M} X_i^k X_i^{k+1} \equiv A(k), \tag{17}$$

for M years of data and here $\Delta t = 1$ month so $k + \Delta t = k + 1$. Thus, $S(t)$ is an estimation of the monthly variance (and hence standard deviation) over the M-year record, and $A(t)$ is the intermonthly correlation between two adjacent months.

We use equation (10) to estimate $\langle f^2\rangle P(t)$ in equation (16) as follows. The solutions of equation (6) converge to a periodically stationary solution when $\int_0^\Gamma a(r)dr < 0$ and hence the annually averaged system is stable. Now, let $\Delta t = m\Gamma$ where m is an integer characterizing an intermediate time scale such that $\mathcal{T}_a \ll m\Gamma \ll 1/\epsilon$, so that $\exp\left(\int_t^{t+m\Gamma} a(r)dr\right) \ll 1$. Now, combining this with the fact that $P(t)$ is periodic with period Γ, equation (10) gives

$$\langle x(t)x(t+m\Gamma)\rangle \simeq \langle f^2\rangle P^2(t). \tag{18}$$

Now we can approximate $\langle x(t)x(t+m\Gamma)\rangle$ from the time series data as

$$\langle x(k)x(k+m\Gamma)\rangle \simeq \frac{1}{M-m-1}\sum_{i=1}^{M-m} X_i^k X_{i+m}^k \equiv B(k), \tag{19}$$

thereby allowing equation (16) to be rewritten as

$$P(t)A(t) = [P(t+\Delta t)-\Delta t]\,S(t) + B(t)\Delta t. \tag{20}$$

Approximating $P(t+\Delta t) \simeq P(t) + \frac{dP}{dt}\Delta t$ leads to the following ordinary differential equation,

$$\frac{dP}{dt} = -\frac{1}{\Delta t}\frac{S(t)-A(t)}{S(t)}P(t) + \frac{S(t)-B(t)}{S(t)}. \tag{21}$$

Defining

$$G(t) \equiv \frac{1}{\Delta t}\frac{S(t)-A(t)}{S(t)} \quad \text{and}$$
$$H(t) \equiv \frac{S(t)-B(t)}{S(t)}, \tag{22}$$

and letting $t = n\Gamma + \tilde{t}$, where $0 < \tilde{t} < 1$, when we take the limit $n \to \infty$ we find

$$P(t) = \exp\left(-\int_0^{\tilde{t}} G(r)dr\right)\left[\int_0^{\tilde{t}} H(r)\exp\left(\int_0^r G(s)ds\right)dr + \frac{1}{e^{\Gamma}-1}\int_0^{\Gamma} H(r)\exp\left(\int_0^r G(s)ds\right)dr\right], \tag{23}$$

where $\Gamma \equiv \int_0^{\Gamma} G(r)dr$. Now, from the definition of $P(t)$ we have

$$\frac{dP(t)}{dt} = a(t)P(t) + 1, \tag{24}$$

and using equation (21) this leads us to

$$a(t)P(t) + 1 = H(t) - G(t)P(t), \tag{25}$$

from which we finally obtain and expression for the stability parameter as

$$a(t) = \frac{1}{P(t)}\left[H(t) - G(t)P(t) - 1\right]. \tag{26}$$

The remaining quantities to be determined are $N(t)$ and $f(\tau)$.

B. Determination of $N(t)$.

We let

$$y(t) = x(t+\Delta t) - x(t) - a(t)x(t)\Delta t, \tag{27}$$

and hence the estimation of $y(t)$ is $N(t)\Delta W + f(\tau)\Delta t$. Thus, the variance of $y(t)$ is

$$\langle y^2(t)\rangle = N^2(t)\Delta t + f^2(\tau)\Delta t^2, \tag{28}$$

and the autocorrelation is

$$\langle y(t)y(t+\Delta t)\rangle \simeq f^2(\tau)\Delta t^2, \tag{29}$$

from which we find

$$N^2(t) = \frac{1}{\Delta t}\left(\langle y^2(t)\rangle - \langle y(t)y(t+\Delta t)\rangle\right). \tag{30}$$

C. Determination of $f(\tau)$.

The final stage is to estimate the value of $f(\tau)$, which is clearly a stochastic variable due to the presence of the noise $N(t)\xi(t)$, but it is a slow variable relative to that noise. Thus, from the stochastic model (equation 6) we write

$$\int_t^{t+\Gamma}\frac{dx(t)}{dt}dt = \int_t^{t+\Gamma} a(t)x(t)dt + \int_t^{t+\Gamma} N(t)dW + f(\tau)\int_t^{t+\Gamma} dt, \tag{31}$$

which leads to

$$f(\tau) = x(t+\Gamma) - x(t) - \int_t^{t+\Gamma} a(t)x dt - \int_0^{\Gamma} N(t)dW, \tag{32}$$

where t is t_k with $1 \le k \le 12$, discretising time in months. Thus we have twelve monthly equations over the total period analysed, and hence we can consider their average as

$$f(\tau) = \frac{1}{n}\sum_{k=1}^{n} x(t_k+\Gamma) - \frac{1}{n}\sum_{k=1}^{n} x(t_k) - \frac{1}{n}\sum_{k=1}^{n}\int_{t_k}^{t_k+\Gamma} a(t)x(t)dt - \frac{1}{n}\sum_{k=1}^{n}\left[\int_0^{\Gamma} N(t)dW\right]_k, \tag{33}$$

where $n = 12$ and the last random term is different for each k. Therefore, over a periodic average of period Γ equal to one year, we can construct $f(\tau)$ with an uncertainty given by the last random term, which is

$$\sigma^2 = \int_0^{\Gamma} N^2(t)dt. \tag{34}$$

Our estimation of $f(\tau)$ is the mean of equation (33), which is

$$\langle f(\tau)\rangle \simeq \frac{1}{n}\sum_{k=1}^{n} x(t_k+\Gamma) - \frac{1}{n}\sum_{k=1}^{n} x(t_k) - \frac{1}{n}\sum_{k=1}^{n}\int_{t_k}^{t_k+\Gamma} a(t)x(t)dt. \tag{35}$$

III. VALIDITY TEST USING AN IDEALISED MODEL

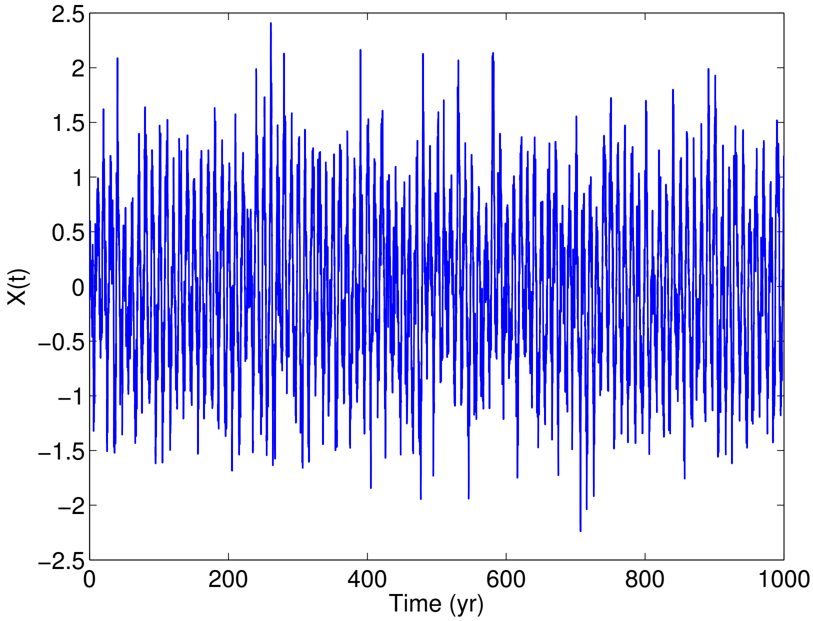

FIG. 1: The surrogate 1000 year time-series generated by equation (36). For parity with the monthly climate data, the basic time step is $\Delta t = 1/12$, and we note that generally speaking for stochastic differential equations one must carefully consider the smallest possible time step by considering issues of convergence and stability [e.g., ref. 1, and textbooks on stochastic differential equations]. For the noise, at each time step we use a Gaussian random variable with zero mean and standard deviation $\sqrt{\Delta t}$. The coefficients $a(t)$ and $N(t)$ have a periodicity of 1 and the long term forcing $f(\tau)$ has a periodicity of 10, thereby capturing decadal forcing.

We test the method using an idealised surrogate data set generated using a simple periodic non-autonomous stochastic model containing long-term forcing, which we write as

$$\frac{dx(t)}{dt} = -[1+\sin(2\pi t)]x(t) + [0.5 + 0.2\cos(2\pi t)]\xi(t) + \cos\left(\frac{2\pi}{10}t\right). \tag{36}$$

Thus, in the notation of the original model equation (6) we have

$$\begin{aligned} a(t) &= -[1+\sin(2\pi t)], \\ N(t) &= 0.5 + 0.2\cos(2\pi t), \quad \text{and} \\ f(\tau) &= \cos\left(\frac{2\pi}{10}t\right), \end{aligned} \tag{37}$$

where the periodicity of $a(t)$ and $N(t)$ is 1, and that of $f(\tau)$ is 10. As also noted in the caption of figure 1, to generate the surrogate data, we evolve equation (36) numerically with a time step of $\Delta t = 1/12$, and at each time step, we use Gaussian noise with zero mean and standard deviation $\sqrt{\Delta t}$.

Figure 1 shows the surrogate data and we use it to deduce $a(t)$, $N(t)$ and $f(\tau)$ with the method developed here. We then compare the values so determined with those of the model, as shown in figures 2 (a), (b) and (c) respectively, and we show the power spectra in figure 2 (d). There is reasonable match between the derived (solid blue lines) and the "observed" (dashed red lines) data, showing that the method developed here works well so long as the time series is driven by processes with distinctly separable time scales. Finally, we examine the effect of the period of $f(\tau)$ in figures 3 and 4, where the periods of $f(\tau)$ are 5.0 and 2.5 respectively. As expected, as the period shortens, the errors in determining $a(t)$ and $N(t)$ increase.

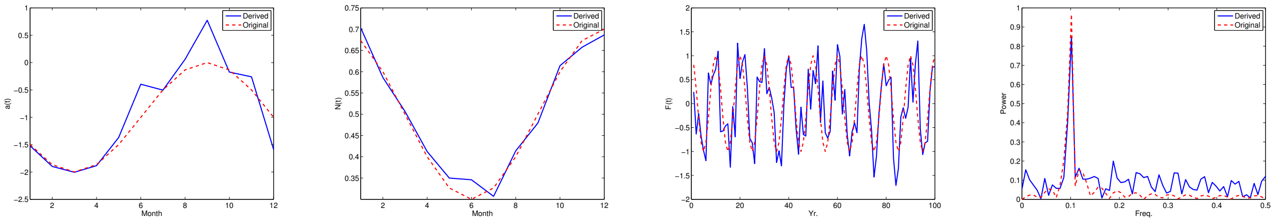

(a) (b) (c) (d)

FIG. 2: The time series of $a(t)$, $N(t)$ and $f(\tau)$ constructed from the method (blue) compared to the surrogate data (red dashed) are shown in (a), (b) and (c) respectively. The power spectra of $f(\tau)$ are compared in (d).

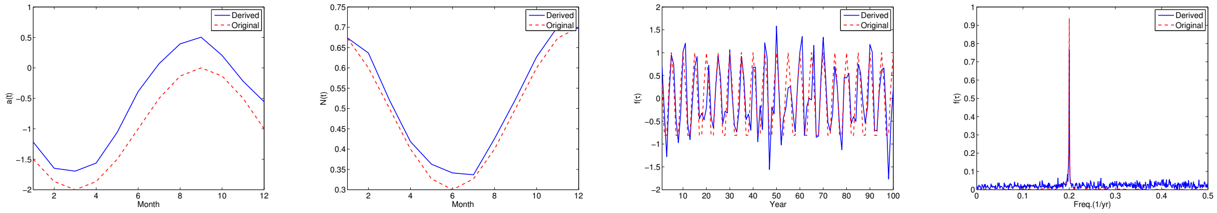

(a) (b) (c) (d)

FIG. 3: The same as figure 2 but the period of $f(\tau)$ is 5.0 instead of 10.0.

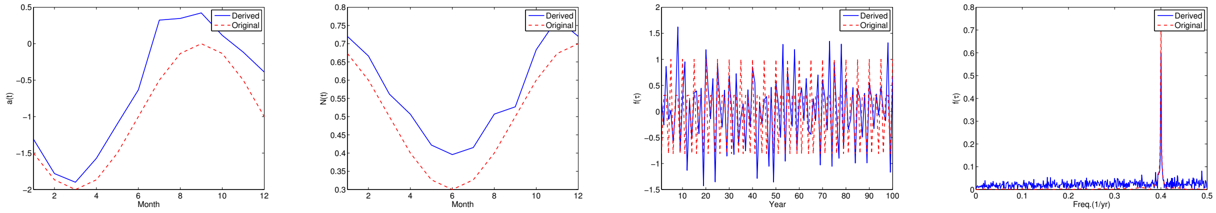

(a) (b) (c) (d)

FIG. 4: The same as figure 2 but the period of $f(\tau)$ is 2.5.

IV. CALCULATION OF PERSISTENCE

The steady-state solution of equation (6) is

$$x(t) = \exp\left(\int_0^t a dr\right) \int_0^t N(t') \exp\left(-\int_0^{t'} a ds\right) dW', \tag{38}$$

which is the first term in equation (7). Therefore, the associated intermonthly autocorrelation function is

$$\begin{aligned}
&\langle x(t)x(t+k)\rangle \\
&= \exp\left(\int_t^{t+k} adr\right)\left\langle\left\{\exp\left(\int_0^t adr\right)\int_0^t N(t')\exp\left(-\int_0^{t'} ads\right)dW'\right\}^2\right\rangle \\
&+\left\langle \exp\left(\int_0^{t+s} adr\right)\int_0^t N(t')\exp\left(-\int_0^{t'} ads\right)dW'\int_t^{t+k} N(t'')\exp\left(-\int_0^{t''} ads\right)dW''\right\rangle, \qquad (39)
\end{aligned}$$

and since

$$\left\langle\left(\int_0^t N(t')\exp\left(-\int_0^{t'} ads\right)dW'\right)\times\left(\int_t^{t+k} N(t'')\exp\left(-\int_0^{t''} ads\right)dW''\right)\right\rangle = 0, \qquad (40)$$

we have

$$\begin{aligned}
&\langle x(t)x(t+k)\rangle \\
&= \exp\left(\int_t^{t+k} adr\right)\exp\left(2\int_0^t adr\right)\int_0^t N^2(t')\exp\left(-2\int_0^{t'} ads\right)dt' \\
&= \exp\left(\int_t^{t+k} adr\right)\langle x^2(t)\rangle. \qquad (41)
\end{aligned}$$

Therefore, the persistence is

$$\frac{\langle x(t)x(t+k)\rangle}{\sqrt{\langle x^2(t)\rangle}\sqrt{\langle x^2(t+k)\rangle}} = \exp\left(\int_t^{t+k} adr\right)\frac{\sqrt{\langle x^2(t)\rangle}}{\sqrt{\langle x^2(t+k)\rangle}}. \qquad (42)$$

V. ANALYSIS OF ERROR

The method relies on $\exp\left(\int_0^\Gamma a(t)dt\right) << 1$ and $\langle f^2\rangle \simeq \langle f(\tau)f(\tau+\Gamma)\rangle$, which leads to the main approximation that $B(t) \equiv \langle x(t)x(t+\Gamma)\rangle \simeq \langle f^2\rangle P^2(t)$. Here we discuss the errors associated with this approximation.

Firstly, without making the above approximation $B(t)$ is

$$\begin{aligned}
B(t) &= e^{-\gamma}(S(t)-\langle f^2\rangle P^2(t)) + \langle f(\tau)f(\tau+\Gamma)\rangle P^2(t) \\
&= e^{-\gamma}(S(t)-\langle f^2\rangle P^2(t)) + (1-\delta)\langle f^2\rangle P^2(t), \qquad (43)
\end{aligned}$$

where $\delta \equiv 1-\frac{\langle f(\tau)f(\tau+\Gamma)\rangle}{\langle f^2\rangle}$, $\exp\left(\int_t^{t+\Gamma} a(r)dr\right) \equiv \mathrm{e}^{-\gamma}$ and hence

$$\langle f^2\rangle P^2(t) = \frac{B(t)-e^{-\gamma}S(t)}{1-\delta-e^{-\gamma}}. \qquad (44)$$

Therefore, equation (21) without the above approximation is

$$\frac{dP}{dt} = -G(t)P(t) + \frac{1}{1-\delta-e^{-\gamma}}H(t) - \frac{\delta}{1-\delta-e^{-\gamma}}, \qquad (45)$$

which has solution

$$P = \frac{1}{1-\delta-e^{-\gamma}}P_0 - \frac{\delta}{1-\delta-e^{-\gamma}}P_1, \qquad (46)$$

where

$$\begin{aligned}
\frac{dP_0}{dt} &= -G(t)P_0(t) + H(t) \quad \text{and} \\
\frac{dP_1}{dt} &= -G(t)P_1(t) + 1. \qquad (47)
\end{aligned}$$

Based on equation (45), we obtain an new version of equation (26) as follows

$$a(t) = -G(t) + \frac{1}{1-\delta-e^{-\gamma}}\frac{H(t)}{P(t)} - \frac{\delta}{1-\delta-e^{-\gamma}}\frac{1}{P(t)} - \frac{1}{P(t)}, \tag{48}$$

which leads to

$$a(t) = -G(t) + \frac{H(t)}{P_0-\delta P_1} - \frac{1-e^{-\gamma}}{P_0-\delta P_1}. \tag{49}$$

Following the assumptions that $\delta << 1$ and $e^{-\gamma} << 1$, we have

$$\begin{aligned} a(t) &= -G(t) + \frac{H(t)}{P_0(t)} - \frac{1}{P_0(t)} - \delta\frac{P_1(t)}{P_0^2(t)}(1-H(t)) + \frac{e^{-\gamma}}{P_0(t)} + \delta e^{-\gamma}\frac{P_1(t)}{P_0^2(t)} \\ &\simeq a_0(t) - \delta\frac{P_1(t)}{P_0^2(t)}(1-H(t)) + \frac{e^{-\gamma}}{P_0(t)}, \end{aligned} \tag{50}$$

in which $a_0(t)$ is that from the approximate method viz., equation (26). Therefore we see that there are two errors associated with the determination of $\langle f^2\rangle P^2(t)$ using the approximate version of $B(t)$. Fortunately, the first correction term is negative and the second correction term is positive, thereby compensating each other. Generally, these two terms are not exactly same in magnitude, which is dependent on the characteristics of a given data. However, we can conclude that the total error is not as large as either one individually.

VI. ONE- VERSUS TWO-DIMENSIONAL STOCHASTIC MODEL FOR ENSO

The core feature of ENSO dynamics is recognized to be inter-annual oscillations principally caused by air-sea interaction. During these stochastic oscillations, the positive and negative extremes of El-Niño and La Niña, are revealed. The intensity of these two phases (positive and negative) is not symmetrical, and hence distributions are not strictly Gaussian, which may be related to multiplicative noise. Our model clearly does not contain these features–mathematically, it is not possible for us to embody both oscillatory and non-Gaussian behavior. We stress that *we do not intend to make the most reliable ENSO model.* Rather, our interest lies on the *seasonal variability of ENSO.*

There are two important behaviors of the seasonal variability of ENSO; (a) the two extreme phases–El Niño and La Niña–are slaved to a specific time of year, and (b) a vast array of prediction models deviate substantially from observations starting in the spring. Using our method, we found the existence of a positive feedback in the eastern Pacific and then we used the interaction of the seasonal stability with the noise to explain the basic mechanism of these two behaviors.

The validity of this argument comes from the fact that the seasonal statistics of the model are very nearly equal to those from the observational data (shown in Fig. 3 in the main text), and the unstable conditions in the eastern Pacific from July to November due to the Bjerknes feedback has been known from previous research. Now, one of the remaining questions is the relationship between our model and the stochastic recharge model. Recently, these research topics–seasonal phase-locking of ENSO and spring predictability barrier–have been investigated using the stochastic recharge model (Refs 34-36 in the main text). Here we show that there is the statistical consistency between our 1D model and the stochastic recharge model in the first two moments *solely in the seasonal time domain.* Moreover, our 1D model shares the same basic seasonal features with the stochastic recharge model used in Refs. 34-36. Hence, the physical mechanism revealed by our model is also contained in the stochastic recharge model.

Here we introduced a simple 1-D periodic non-autonomous stochastic model to simulate, among other things, the seasonal variability of ENSO. This may be considered as far simpler than, or unrealistic relative to, the so-called recharge oscillator model [2], which is a 2-D dynamical system describing the interaction between the sea surface temperature T and the thermocline depth h. In order to generate a spring predictability barrier, the original autonomous model has been modified to include the seasonality of the ENSO growth rate [3]. Here we show that the $a(t)$ derived in our time-series method is equivalent to ENSO's growth rate in the non-autonomous recharge oscillator model, which is

$$\begin{aligned} \frac{dT}{dt} &= a(t)T + N(t)\xi + \omega_E h \quad \text{and} \\ \frac{dh}{dt} &= -\omega_E T, \end{aligned} \tag{51}$$

where $\omega_E = \pi/2$, and

$$a(t) = -2.0 - 2.5\sin(\omega_A t) \quad \text{and}$$
$$N(t) = \ 1.0 + 0.2\sin(\omega_A t), \tag{52}$$

with ω_A the frequency of the seasonal cycle. The functional forms for $a(t)$ and the values of ω_E are exactly same as those used by Levine and McPhaden [3]. The noise $N(t)$ is based upon that derived from the NINO3 index.

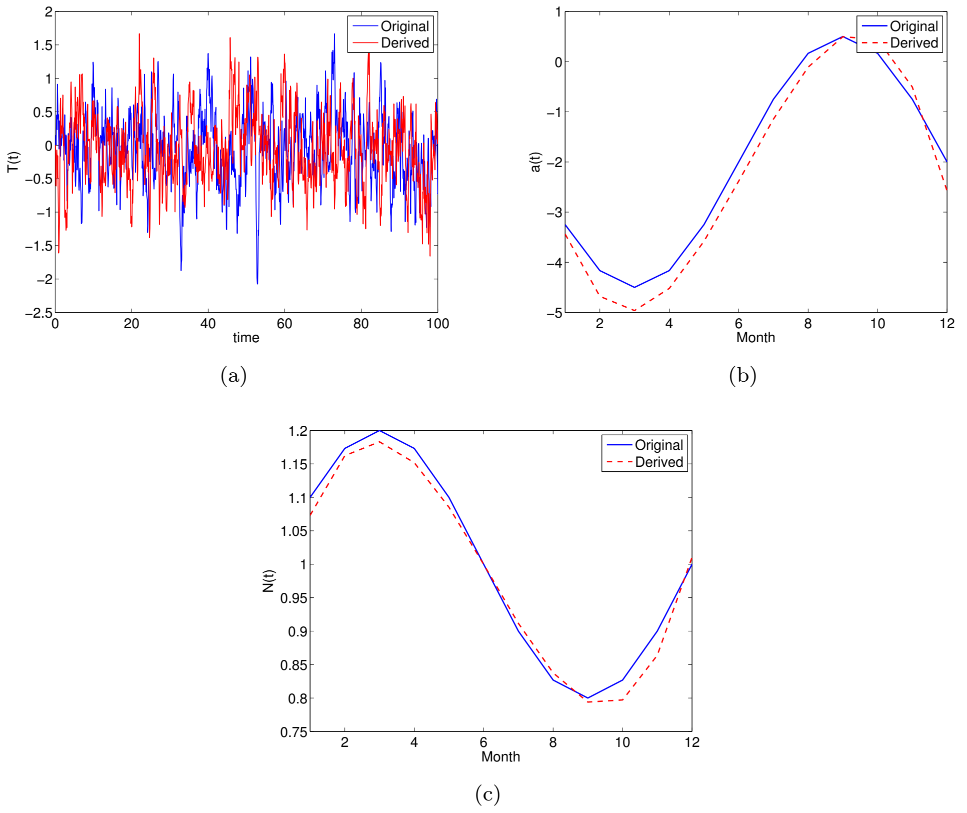

FIG. 5: (a) The surrogate time-series from the stochastic recharge oscillator model of equations (51) and (52) is compared with that from the model $\dot{x}(t) = a(t)x(t) + N(t)\xi(t)$, where $a(t)$ and $N(t)$ are constructed using our time series method and treating the recharge oscillator model as data. In (b) and (c) the $a(t)$ and $N(t)$ from equations (52) are compared with those constructed using our time series method.

A surrogate time-series for $T(t)$ is generated using equations (51) and (52) above with a time-step of 1/12 and spanning a $1000 - yr$ time period. The data so generated is then analysed by our time-series method from which we extract $a(t)$ and $N(t)$. We then compare the results from the recharge oscillator model of equations (51) and (52) to the model $\dot{x}(t) = a(t)x(t) + N(t)\xi(t)$, in which $a(t)$ and $N(t)$ are extracted from our time series approach. In Fig. 5(a) the two time series are shown and the $a(t)$ and $N(t)$ are compared in (b) and (c), respectively. We see that the $a(t)$ and $N(t)$ extracted from our method nearly exactly follow the original $a(t)$ and $N(t)$ of equations (52) used in the stochastic recharge oscillator model. Thus, even though the surrogate data originates from a 2-D dynamical system, we find the ENSO growth rate $a(t)$ and the noise intensity $N(t)$ from the method based on 1-D model. Now we ask why this dimensional reduction is possible?

The model $\dot{x}(t) = a(t)x(t) + N(t)\xi(t)$ represents the seasonal variability, within which the positive/negative phases of ENSO are interpreted, rather than solely in terms of oscillatory behavior. Because of the large magnitude of the variability that is intrinsically represented in the model as El Niño and La Niña, they (i.e., the phases) are not distinguished within it. In the recharge model, the oscillatory interaction between T and h is entirely characterized by the time-scale ω_E, thereby distinguishing the positive or negative phase of ENSO.

The span of the NINO3 index data provides 145 seasonal cycles. In the original time-series there exists an oscillatory behaviour with an inter-decadal time scale. Now, if we randomly shuffle the 145 seasonal cycles, and then re-order the original time series, we would lose the oscillatory behaviour. Therefore, the randomly shuffled time series only contains the principal characteristics of the seasonal dynamics. Hence, regardless of the phase of ENSO, we will still

observe the maximal variance near the end of year due to the memory effect controlled by the positive feedbacks from July to November, thereby capturing the central physical processes that produce *either* phase of ENSO. Therefore, because the two models share the main physics that controls the seasonal variability of ENSO, they also share the same $a(t)$ and $N(t)$, as shown in Fig. 5.

VII. ADVECTION AND THE LOCAL ENERGY FLUX BALANCE MODEL

We analyze local temperature data using this time-series method to study the seasonal characteristics of local climate. Hence, here we clarify how our model treats the contribution of advection at local points, which can play an important role in controlling seasonal climate variability. To this end, we return to equation (3) above ignoring the noise viz.,

$$\frac{dT}{dt} = Q(t,T) + F(\tau), \tag{53}$$

and we show how the noise term, which captures the high frequency effects of weather, emerges naturally from our derivation in the same general (but not formal) sense as in stochastic mode reduction [4].

Whereas for hemispheric, or globally averaged, temperature, the advective contribution to d/dt can be ignored because the domain is large and closed. However, at a local point we consider advection in the usual manner as

$$\frac{\partial T}{\partial t} + u\frac{\partial T}{\partial x} + v\frac{\partial T}{\partial y} = Q(t,T) + F(\tau), \tag{54}$$

where $T = \bar{T} + x'$, $u = \bar{u} + u'$ and $v = \bar{v} + v'$, in the sense of Reynolds decomposition; the overbar denotes the monthly average and x', u' and v' are the deviations from those averages. The central concept is the time-scale separation between the mean and the deviation underlying the Reynolds decomposition. We use a month for time averaging, in which case the deviations represent the contribution of synoptic dynamical phenomena. The mathematical terms containing the deviations are related to the so-called "eddy-mean" or "eddy-eddy" interactions. Intuitively, weather phenomena induced mainly by synoptic dynamics are highly nonlinear and chaotic. Due to the variety of phenomena that collectively represent these dynamics, and their highly efficient mixing characteristics, the overall dynamical and energetic effects from these chaotic dynamics can be approximated as a noise term on the monthly time-scale. Importantly, in equations (56-58) only, x' is equivalent to x in the rest of the manuscript, but for the immediate development we must distinguish between the temperature deviations and the spatial coordinate. Here the monthly average quantities satisfy

$$\frac{\partial \bar{T}}{\partial t} + \bar{u}\frac{\partial \bar{T}}{\partial x} + \bar{v}\frac{\partial \bar{T}}{\partial y} = Q(t,\bar{T}) + \bar{F}, \tag{55}$$

and hence the evolution equation for the temperature deviation x' is

$$\frac{\partial x'}{\partial t} + u'\frac{\partial \bar{T}}{\partial x} + v'\frac{\partial \bar{T}}{\partial y} + \bar{u}\frac{\partial x'}{\partial x} + \bar{v}\frac{\partial x'}{\partial y} + u'\frac{\partial x'}{\partial x} + v'\frac{\partial x'}{\partial y} = a(t)x' + f(\tau). \tag{56}$$

Because we are concerned with seasonal time scales, the derivative $\partial/\partial t$ is characterized by a time scale of $\sim$ one month. The advection of the deviations, or anomalous quantities, is due to the quasi-geostrophic dynamics which govern weather. Thus, the length scale characterizing advection is synoptic and $\sim$ 1000 km, or the atmospheric Rossby radius of deformation. Because the typical velocity scale is $\sim 10\text{m s}^{-1}$, then the advection time-scale approximately a day. Therefore, there is large separation between the time scale characterizing $\partial/\partial t$ and that characterizing advection, which is why we treat the weather as having a short memory and write

$$u'\frac{\partial \bar{T}}{\partial x} + v'\frac{\partial \bar{T}}{\partial y} + \bar{u}\frac{\partial x'}{\partial x} + \bar{v}\frac{\partial x'}{\partial y} + u'\frac{\partial x'}{\partial x} + v'\frac{\partial x'}{\partial y} \simeq -N(t)\xi. \tag{57}$$

Finally, letting $x' \to x$, the local temperature fluctuations are approximately represented by

$$\frac{dx}{dt} = a(t)x(t) + N(t)\xi(t) + f(\tau). \tag{58}$$

Clearly then, as shown in this derivation, our method does not ignore the contribution of local advection. The mean advection of the temperature is captured by its average seasonal cycle, which is the signal against which the relative dynamics is assessed, that is, the seasonal cycle is removed from the dynamics. Thus, the advection of x is contained in the noise term $N(t)\xi(t)$, and the equation above is the same as that derived in §I and the discussion surrounding Eq. (1) of the main text.

VIII. SAMPLING ERRORS IN $a(t)$ AND $N(t)$

The error in all statistical methods depends on the number of samples, although there are many model specific ways to treat errors depending on the specific system under consideration. The solution of the main linear stochastic differential equation is

$$x(t) = x(0)\exp\left(\int_0^t a dr\right)\int_0^t N(t')\exp\left(-\int_0^{t'} a ds\right) dW' + f(\tau)P(t), \tag{59}$$

where $f(\tau) = \langle f(\tau)\rangle + \sigma\xi$, ξ is white noise, and $\sigma = \sqrt{\int_0^\Gamma N^2(t)dt}$, which is derived in equation (34) above. We can determine $\langle f(\tau)\rangle$ using a moving average or a high-frequency filter based on the Fast Fourier Transform (FFT). Now, we introduce a new stochastic variable $Z(t) \equiv x(t) - \langle f(\tau)\rangle\langle P(t)\rangle$, where $Z(t)$ is assumed to have short-time-scale dynamics. Hence $\langle Z(t)Z(t+m\Gamma)\rangle = 0$, with $\Gamma = 1$yr and m a positive integer, which is equivalent to defining the term $B(t) \equiv \langle x(t)x(t+m\Gamma)\rangle = \langle f^2(\tau)\rangle P^2(t)$. We can deduce that the sampling error of the mean of the stochastic variable $Z(t)Z(t+m\Gamma)$ is proportional to that of $\langle P(t)\rangle$, which leads to

$$P'(t) \sim \mathcal{N}\left(0, \frac{1}{4\langle f^2\rangle^2\langle P(t)\rangle^2}\frac{\sigma_z^2}{n}\right), \tag{60}$$

where $\mathcal{N}$ denotes the normal distribution, σ_z the standard deviation of the stochastic variable $Z(t)Z(t+m\Gamma)$ and n the sample number, which in our case is the total number of years in the data set. Thus, $P(t)$ satisfies

$$\frac{dP}{dt} = a(t)P(t) + 1, \tag{61}$$

which leads to

$$P(t+\Delta t) = (1 + a(t)\Delta t)P(t) + \Delta t. \tag{62}$$

The relationship between the sampling errors in $P(t)$ and $a(t)$ is

$$a'(t) = \frac{1}{\Delta t\langle P(t)\rangle}\left(P'(t+\Delta t) - (1+\langle a\rangle\Delta t)P'(t)\right), \tag{63}$$

and hence the sampling error of $a(t)$ is

$$\langle a'^2(t)\rangle = \frac{1}{\Delta t^2\langle P(t)\rangle^2}\left(\langle P'^2(t+\Delta t)\rangle + (1+\langle a\rangle\Delta t)^2\langle P'^2(t)\rangle\right). \tag{64}$$

Next we derive the sampling error of the noise intensity $N(t)$. The variance of the stochastic variable $Z(t)$ satisfies

$$\frac{d}{dt}\langle Z^2(t)\rangle = 2a(t)\langle Z^2(t)\rangle + N^2(t), \tag{65}$$

which leads to

$$\langle Z^2(t+\Delta t)\rangle - \langle Z^2(t)\rangle = 2a(t)\Delta t\langle Z^2(t)\rangle + N^2(t)\Delta t. \tag{66}$$

Let $\zeta(t)$ be the sampling error of $\langle Z^2\rangle$, which is known to be [5]

$$\sqrt{\langle\zeta^2(t)\rangle} = \langle Z^2\rangle\sqrt{\frac{2}{n-1}}. \tag{67}$$

The sampling errors ζ, a' and N' are interrelated as follows;

$$\zeta(t+\Delta t) - (1 + 2\langle a(t)\rangle\Delta t)\zeta(t) - 2\Delta t\langle Z^2(t)\rangle a'(t) = 2\Delta t\langle N(t)\rangle N'(t), \tag{68}$$

and thus, assuming that ζ and a' are statistically independent, we obtain

$$\langle N'^2(t)\rangle = \frac{1}{4\Delta t^2\langle N(t)\rangle^2}\left(\langle\zeta^2(t+\Delta t)\rangle + (1+2\langle a(t)\rangle\Delta t)^2\langle\zeta^2(t)\rangle + 4\Delta t^2\langle Z^2(t)\rangle^2\langle a'^2(t)\rangle\right). \tag{69}$$

We use the above analysis to assess the sample errors in the GISS Northern hemisphere and Southern hemisphere averaged temperature data. In figure (6) the errors in $a(t)$ and $N(t)$ are shown with blue error bars. Figures 6 (a) and (b) show the Northern hemisphere and (c) and (d) show the Southern hemisphere.

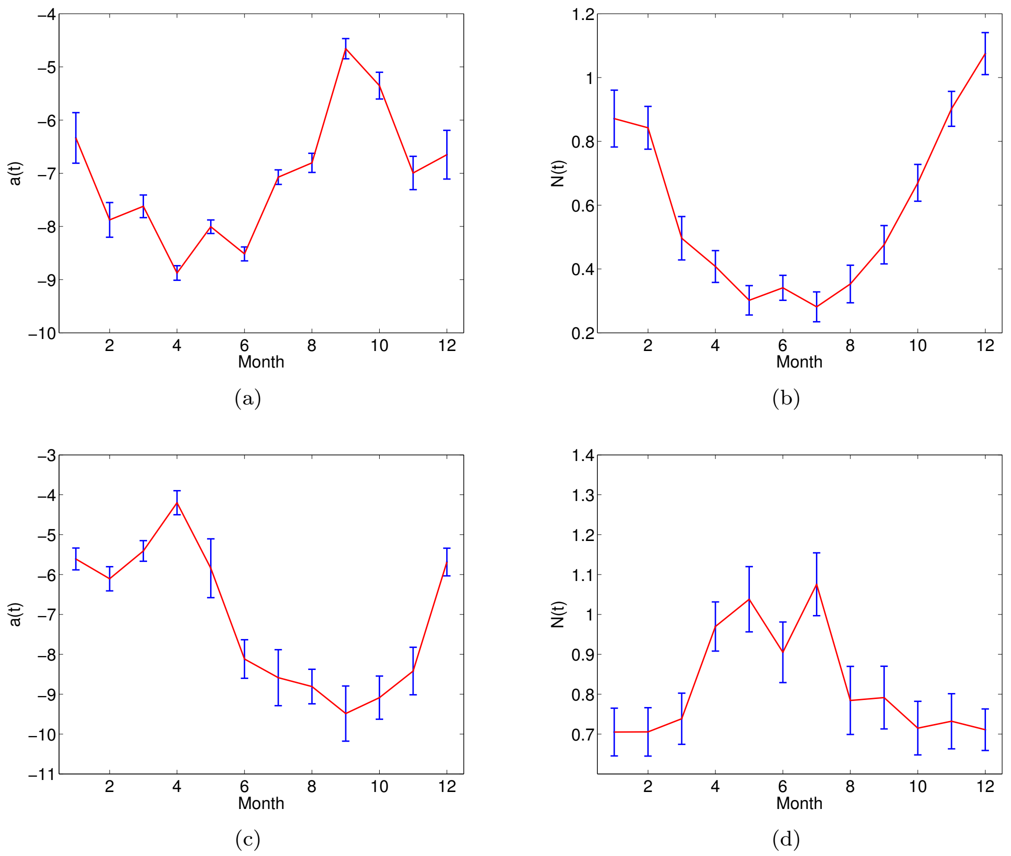

FIG. 6: The sampling errors for $a(t)$ and $N(t)$ for the GISS Northern hemisphere, (a) and (b), and Southern hemisphere, (c) and (d), temperature data.

[1] Moon, W. & Wettlaufer, J. S. A stochastic perturbation theory for non-autonomous systems. *J. Math. Phys.*, **54 (12)**, 123 303 (2013).
[2] Jin, F. F. An equatorial ocean recharge paradigm for ENSO. Part 1: Conceptual model, *J. Atmos. Sci.*, **64**, 811–829 (1997).
[3] Levine, A. F. Z. & McPhaden, M. J. The annual cycle in ENSO growth rate as a cause of the spring predictability barrier, *Geophys. Res. Lett.*, **42**, 5034–5041(2015).
[4] Majda, A. J. *Introduction to Turbulent Dynamical Systems in Complex Systems, Springer – Frontiers in Applied Dynamical Systems: Reviews and Tutorials* (Springer, Switzerland) (2016).
[5] Ahn, S., & Fessler, J. A. Standard errors of mean, variance and standard deviation estimators, *University of Michigan: EECS Department* (2003).
